# Educational Case: Squamous cell carcinoma

**DOI:** 10.1016/j.acpath.2025.100206

**Published:** 2025-07-04

**Authors:** Ashley R. Scholl, Melina B. Flanagan, Andrew D. Thompson

**Affiliations:** a Duke University Medical Center, Department of Pathology, DUMC Department of Pathology, Durham, NC, USA; b West Virginia University, Department of Pathology, Anatomy and Laboratory Medicine, Morgantown, WV, USA; c CND Life Sciences, Scottsdale, AZ, USA

**Keywords:** Pathology competencies, organ system pathology, skin, malignant skin neoplasms, squamous cell carcinoma

## Primary objective:

Objective SK5.2: Malignant Skin Neoplasms. Describe the clinical presentation, precursor lesions, risk factors, and hereditary cancer syndromes that lead to the following skin cancers: basal cell carcinomas, squamous cell carcinoma, and melanoma.

The following fictional case is intended as a learning tool within the Pathology Competencies for Medical Education (PCME), a set of national standards for teaching pathology. These are divided into three basic competencies: Disease Mechanisms and Processes, Organ System Pathology, and Diagnostic Medicine and Therapeutic Pathology. For additional information, and a full list of learning objectives for all three competencies, see https://doi.org/10.1016/j.acpath.2023.100086.^1^

Competency 2: Organ System Pathology, Topic: Skin (SK), Learning Goal 5: Skin Neoplasia

## Secondary objectives:

Objective SK5.3: Genetic Disorders Predisposing to Skin Cancer. Identify the genetic disorders with high risk of skin cancers and explain the molecular basis of that risk as well as the genomic mutations involved.

Competency 2: Organ System Pathology, Topic: Skin (SK), Learning Goal 5: Skin Neoplasia

Objective SK5.4: Sun Exposure. Explain the role of ultraviolet light and other environmental factors in development of various skin cancers.

Competency 2: Organ System Pathology, Topic: Skin (SK), Learning Goal 5: Skin Neoplasia

Objective N3.1: Morphologic Features of Neoplasia. Describe the essential morphologic features of neoplasms and indicate how these can be used to diagnose, classify, and predict biological behavior of cancers.

Competency 1: Disease Mechanisms and Processes, Topic: Neoplasia (N), Learning Goal 3: Characteristics of Neoplasia

## Patient presentation

A 70-year-old man presents to his physician with a skin lesion on his forehead. The lesion is particularly bothersome as it has been growing and spontaneously bleeding for the past few months. The patient is a welder and owns his own farm. He is physically active, takes no medications, and has a 50-pack-year smoking history.

## Diagnostic findings, Part 1

The patient is well-appearing and has fair skin. Skin examination reveals numerous scaly white plaques on the dorsal aspect of the hands and forearms. There is a 2 cm, scaly, nonpigmented nodule on the left forehead that bleeds when touched. There are multiple uniformly appearing nevi scattered across his arms, chest, face, and legs. No other worrisome skin lesions are identified, and the remainder of the physical exam is unremarkable.

## Questions/discussion points, Part 1

### Based on the patient's history and the physical exam, what are the main differential diagnoses to consider?

Given the patient's age, fair skin and significant UV exposure (obtained through his occupations as a welder and farmer), the top differential for a nonpigmented lesion is a primary skin cancer such as squamous cell carcinoma, basal cell carcinoma or amelanotic melanoma. The risk of all three of these skin cancers is increased with increased UV light exposure.^2^ Melanomas are typically pigmented lesions (although there are amelanotic melanomas), and our patient's lesion is nonpigmented, which makes a diagnosis of melanoma less likely. Although the patient does not have any significant medical history, another differential to consider would be a cutaneous metastasis from an unknown primary malignancy.

### What is the next step in management of the patient?

To determine what is causing this patient's skin lesion, a sample must be taken and sent to pathology. Because a neoplastic process is favored, the patient undergoes a shave biopsy. Punch biopsies are generally preferred when the clinical suspicion is for an inflammatory process.

## Diagnostic findings, Part 2

**Fig. 1:**
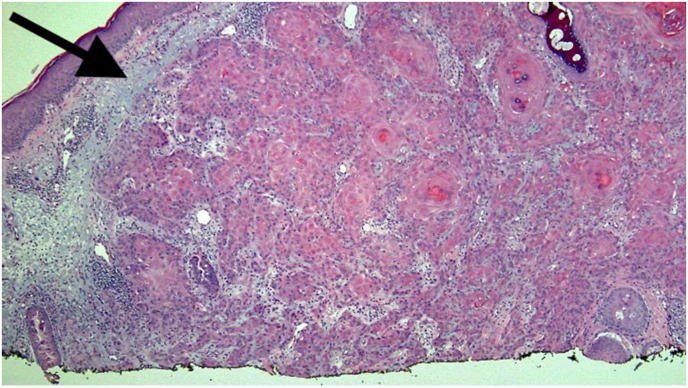
H&E, 40x. This is a shave biopsy demonstrating solar elastosis (black arrow) and an epithelial proliferation in the dermis.

**Fig. 2:**
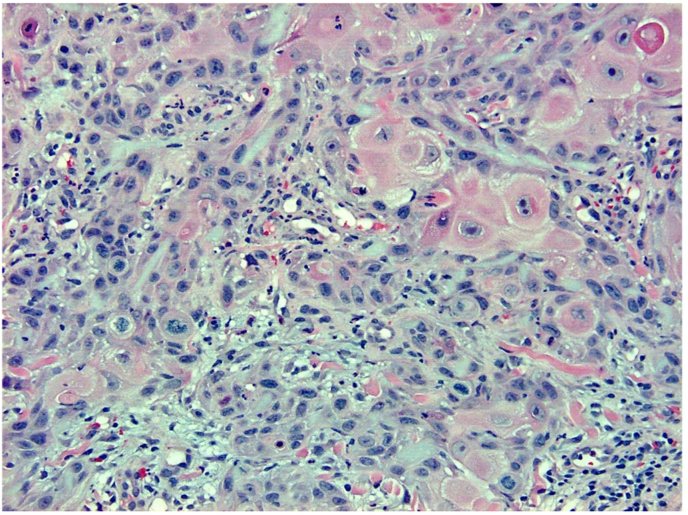
H&E, 200x. This is a biopsy demonstrating neoplastic cells with abundant eosinophilic cytoplasm, prominent nucleoli, and large, pleomorphic nuclei.

**Fig. 3:**
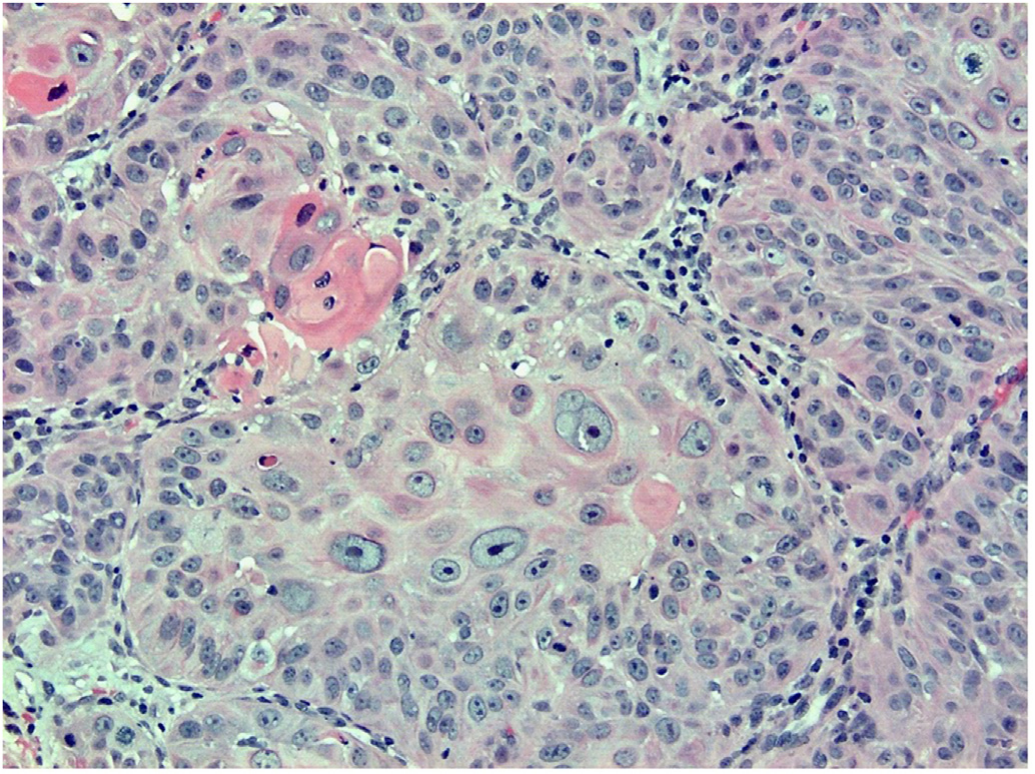
H&E. 200x. This is a biopsy demonstrating neoplastic cells with keratin formation deep in the dermis.

**Fig. 4:**
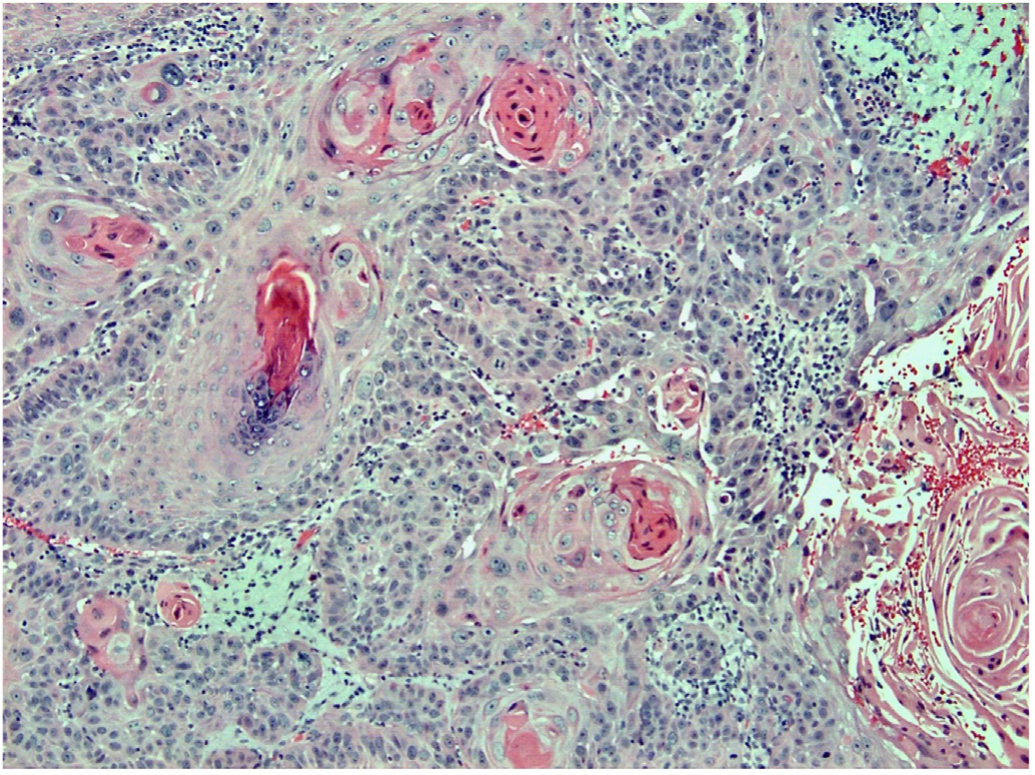
H&E, 100x. Numerous keratin pearls are present.

**Fig. 5:**
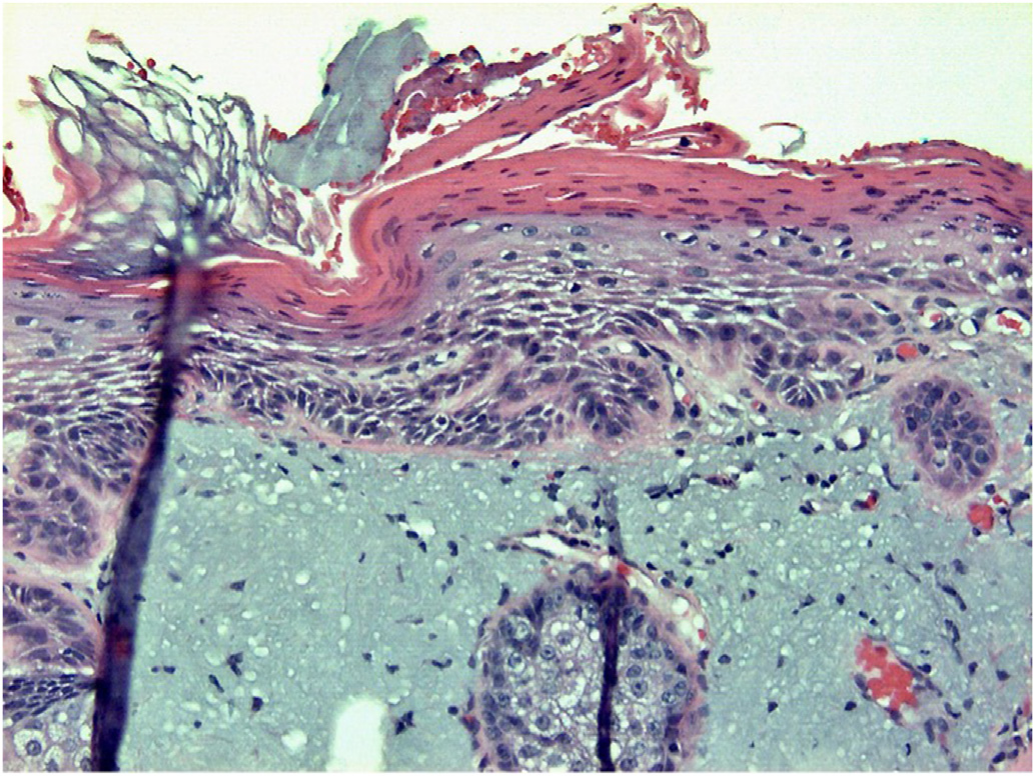
H&E, 200x. This biopsy demonstrates extensively sun-damaged skin, as evidenced by solar elastosis.

Representative photomicrographs from the patient's biopsy are shown in Fig. 1, Fig. 2, Fig. 3, Fig. 4, Fig. 5.

## Questions/discussion points, Part 2

### Describe the histologic findings from the patient's biopsy. What is the diagnosis?

A histologic image of the entire biopsy is shown in Fig. 1. The biopsy shows sheets and nests of atypical keratinocytes that are invading through the dermis. The neoplastic cells can be defined as keratinocytes due to the presence of intercellular attachments (desmosomes) between cells.^3^ The keratinocytes have abundant eosinophilic cytoplasm, prominent nucleoli, and large, pleomorphic nuclei (Fig. 2, Fig. 3). There are areas where the neoplastic keratinocytes have produced an abundance of keratin, called keratin pearls (Fig. 4). The skin also shows significant sun damage, as evidenced by solar elastosis, a fibrillary gray material in the upper dermis (Fig. 5).^4^

Based on the findings in this biopsy specimen, the diagnosis is invasive squamous cell carcinoma.

### In this patient, can a basal cell carcinoma be excluded as a diagnostic consideration?

**Fig. 6:**
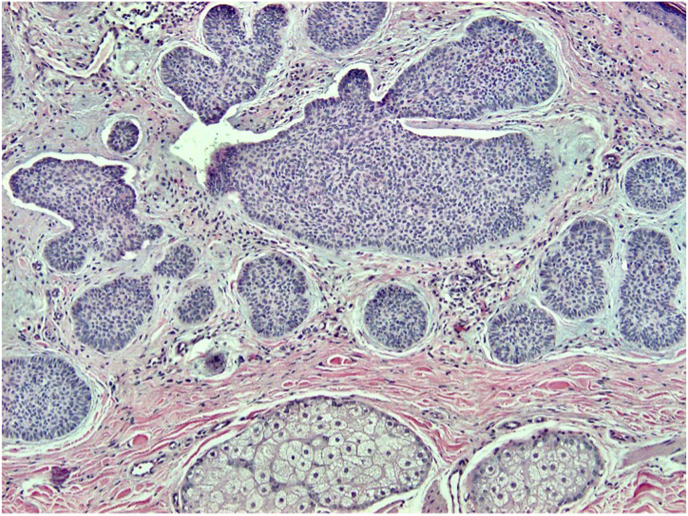
H&E, 100x. Biopsy demonstrating basal cell carcinoma. Basaloid tumor cells are arranged in nests with peripheral palisading of cells, with a mucinous stroma, and retraction of tumor cells away from the stroma. Normal sebaceous glands are present in the lower portion of the image.

Basal cell carcinoma is the most common primary skin malignancy.^2^ It is an important diagnosis to consider in a patient with this presentation. Basal cell carcinomas are also related to UV exposure and are common on the head and neck.^5^^,^^6^ They clinically present as a pearly papule with prominent telangiectasias.^5^ Histologically, they are comprised of darkly hematoxylin-staining (basaloid) cells arranged in nests and nodules, with a palisading of tumor cells around the edges of the nests (Fig. 6).^7^ Basal cell carcinomas show numerous mitoses, apoptotic cells, mucin and a retraction of the tumor cells away from the stroma (Fig. 6).^7^

### Is there an immunohistochemical stain that can help us delineate between squamous cell carcinoma and basal cell carcinoma?

Lesions often show features that overlap with multiple different diagnoses, and in such situations, immunohistochemistry can be helpful in establishing the correct diagnosis. In a lesion that shows features of both basal cell carcinoma and squamous cell carcinoma, the monoclonal antibody Ber-EP4 can be used. It is almost always positive in basal cell carcinoma and negative in squamous cell carcinoma.^8^ Additionally, epithelial membrane antigen (EMA) is an immunohistochemical stain that has a high rate of positivity in squamous cell carcinomas and a low rate of positivity in basal cell carcinomas, making it useful in differentiating between these two diagnoses.^9^

### What is the next step for the patient?

Now that a diagnosis of invasive squamous cell carcinoma has been made, the patient needs to have the remainder of the lesion excised with negative margins. Additionally, a physical exam checking for lymphadenopathy is warranted.

## Diagnostic findings, Part 3

The patient has the remainder of the tumor excised, with negative margins. No lymphadenopathy is found on physical exam.

## Questions/discussion points, Part 3

### How does UV light induce skin damage?

Ultraviolet (UV) light is primarily composed of UVA and UVB, both of which can both damage DNA, either indirectly through the formation of reactive oxygen species, or directly via base transitions.^10^ Normally, the induction of skin apoptosis via sunburn acts as the body's defense mechanism against sustained UV damage.^10^

The mutations caused by UVA and UVB can cause tumor suppressor gene inactivation and oncogene activation. P53 is a tumor suppressor that activates cell cycle inhibitors and apoptosis regulators, and it is directly damaged by UV radiation, which contributes to tumorigenesis.^10^

### What environmental factors lead to the development of squamous cell carcinoma?

The most important modifiable risk factor for the development of cutaneous squamous cell carcinoma is cumulative UV light exposure.^2^^,^^11^ Immunosuppression also carries a higher risk of squamous cell carcinoma. This is particularly relevant in patients who have received an organ transplant or those with a hematolymphoid neoplasm.^11^ Patients on long-term immunosuppression have decreased immune-mediated tumor surveillance, increased susceptibility to oncogenic viruses, and deal with the direct consequences of immunosuppressive agents on initiation of cutaneous cancers.^12^

Additional risk factors include HIV, high levels of arsenic exposure and chronic wounds.^13^^,^^14^^,^^15^

### What are the precursor lesions to squamous cell carcinoma?

**Fig. 7:**
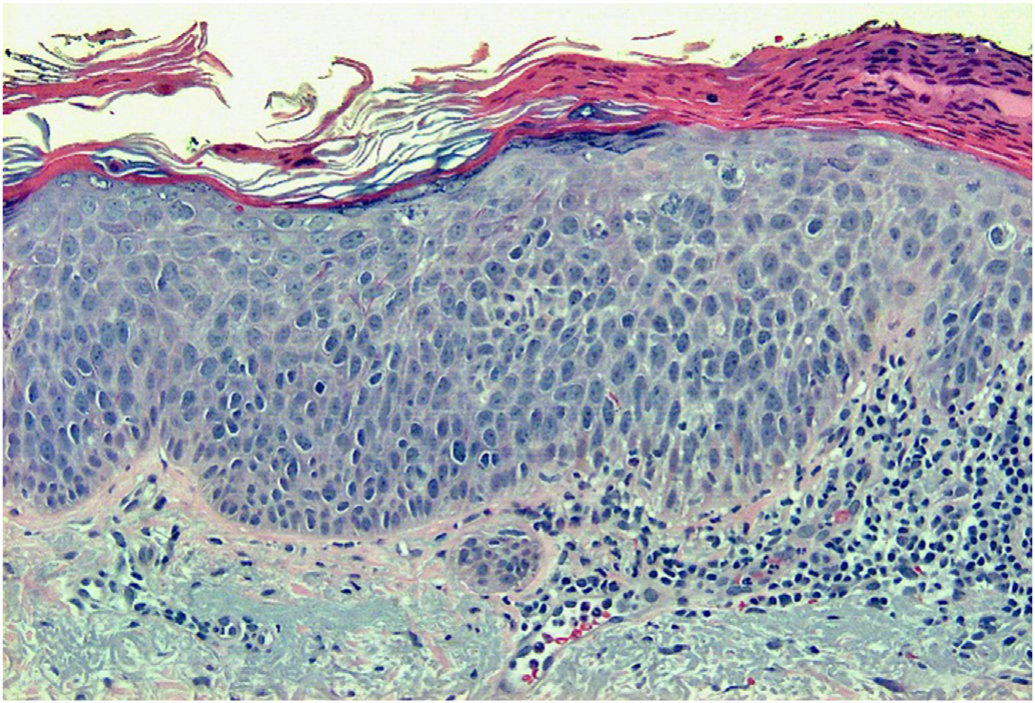
H&E, 200x. Biopsy demonstrating squamous cell carcinoma in situ. Atypical keratinocytes extend full-thickness throughout the epidermis. Note the solar elastosis and chronic inflammation in the dermis.

**Fig. 8:**
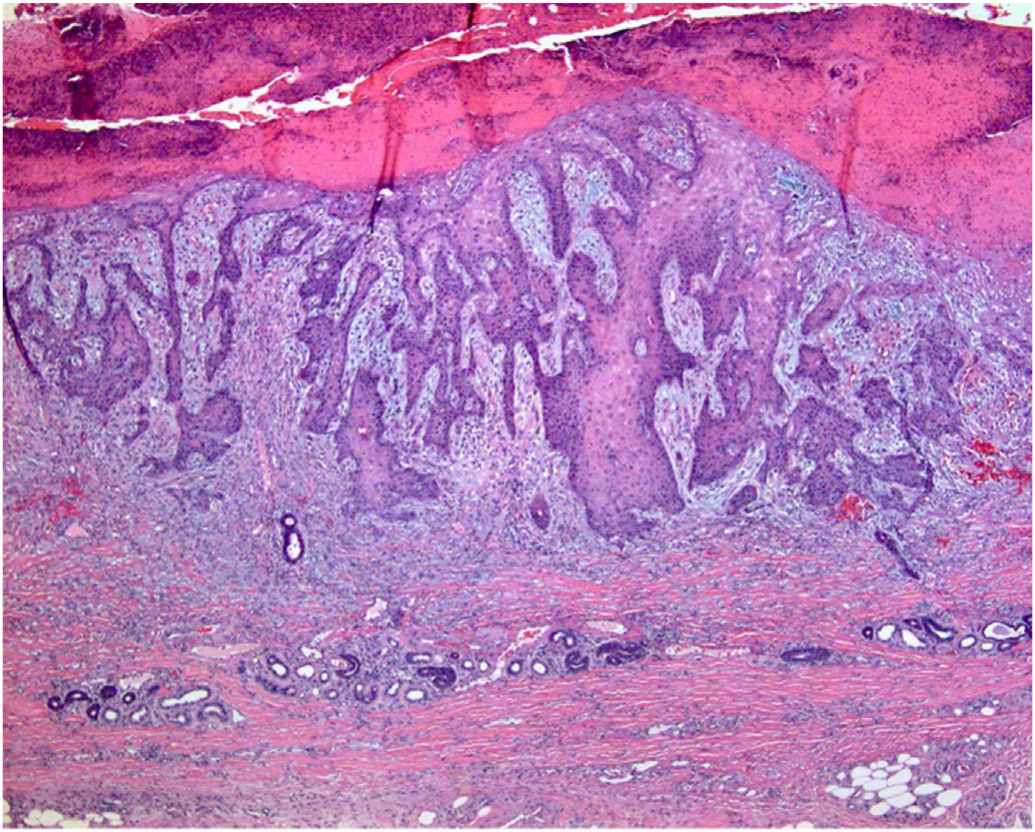
H&E, 40x. This is a biopsy demonstrating invasive squamous cell carcinoma with atypical keratinocytes invading into the dermis.

When the keratinocytes in sun-damaged skin begin to become atypical, the lesion is termed actinic keratosis. If the atypical keratinocytes spread through the entire epidermis (full-thickness atypia) but are still contained within the basement membrane, the lesion is called squamous cell carcinoma in situ (Fig. 7). When the atypical cells break through the basement membrane and invade into the dermis, invasive squamous cell carcinoma is diagnosed (Fig. 8).^11^

### What conditions are associated with multiple basal cell carcinomas and what genetic mutations are implicated?

Gorlin syndrome is an autosomal dominant disorder characterized by mutations in the hedgehog signaling pathway.^16^ The hedgehog signaling pathway is crucial for numerous tissue homeostatic and developmental processes and when altered by mutations, the unregulated activation of this pathway has been demonstrated to be the key molecular driver in the development of these tumors.^17^ Clinically, patients develop multiple basal cell carcinomas in childhood.^18^ A mutation in the tumor suppressor gene, *PTCH1*, is present in approximately 70% of patients with this syndrome.^19^^,^^20^

Xeroderma pigmentosum is an autosomal recessive condition caused by mutations in *XPA-XPF* genes that are involved in nuclear excision repair of DNA damaged by UV radiation.^21^ These patients have 10,000 times the risk of developing nonmelanotic skin cancer as compared to the general population.^21^ The exaggerated response to UV damage leads to premature skin aging and early development of squamous cell carcinoma, basal cell carcinoma, and malignant melanoma, with many patients experiencing their first skin cancer around age nine.^21^

### What are the risk factors, clinical presentation and precursor lesions of melanoma?

Melanoma is a cancer derived from pigment-producing melanocytes in the skin.^22^ Ultraviolet radiation, light skin, a history of sunburns, high numbers of nevi, and a family history of melanoma are all risk factors for the development of invasive melanoma.^22^ Patients usually present with a pigmented lesion on the skin.^23^ Benign and large congenital nevi can gain genetic mutations, which can cause a previously benign lesion to progress to a dysplastic nevus and then to melanoma.^24^ Patients with germline mutations in the tumor suppressor gene *CDKN2A* are at an increased risk of developing melanoma at some point in their lifetime.^22^

### What important preventive measures should be recommended to the patient?

This patient should be wearing sunscreen each day and should wear the appropriate personal protective equipment (PPE) while working as a welder and farmer. Additionally, avoiding sun exposure during peak sunlight hours, wearing sun protective clothing, and seeking shade are recommended to reduce his risk of additional skin cancers.^2^

## Teaching points

• Squamous cell carcinoma, basal cell carcinoma, and melanoma are cutaneous skin cancers that are associated with UV light exposure. The risk of squamous cell carcinoma is increased in patients with organ transplantation or with hematolymphoid neoplasms.
• UV damage causes tumor suppressor gene inactivation and oncogene activation.
• Xeroderma pigmentosum is caused by a mutation in nuclear excision repair of DNA damaged by UV radiation.
• Basal cell carcinoma is the most common skin malignancy and, in rare cases, can be associated with Gorlin syndrome (multiple basal cell carcinomas at an early age). Mutations in the *PTCH1* gene cause unregulated activation of the hedgehog signalizing pathway, which significantly contributes to tumor development.
• Additional risk factors for the development of melanoma include light skin, a history of sunburns, a family history of melanoma, and numerous nevi. Patients with melanoma usually present with a pigmented skin lesion. Germline mutations in *CDKN2A* can cause a hereditary form of melanoma.
• The precursor lesions to invasive squamous cell carcinoma are actinic keratosis and squamous cell carcinoma in situ.
• Patients should be counseled to wear sunscreen and sun protective clothing, avoid peak sunlight hours, and make sure to mitigate occupational risks.

## Funding

The article processing fee for this article was funded by an Open Access Award given by the Society of ‘67, which supports the mission of the Association for Academic Pathology to produce the next generation of outstanding investigators and educational scholars in the field of pathology. This award helps to promote the publication of high-quality original scholarship in *Academic Pathology* by authors at an early stage of academic development.

## Declaration of competing interest

The authors declare that they have no known competing financial interests or personal relationships that could have appeared to influence the work reported in this paper.
